# Patterns and Frequency of Opioid Use Among Individuals With a History of Gastrointestinal Malignancy: A Cross-Sectional Analysis of the 2019-2020 National Health Interview Survey (NHIS)

**DOI:** 10.7759/cureus.115424

**Published:** 2026-08-30

**Authors:** Mohammed D Al-Hejaili, Rami Saleh, Ghazi H Mogharbel, Dana F Maufa, Abdulmohsen Hassan, Maisa Manfaluti, Rifq S Altamimi, Linah A Murtadha, Abdulrahman I Almarwani, Renad K Alandejane

**Affiliations:** 1 Anesthesiology, National Guard Health Affairs, Medina, SAU; 2 Internal Medicine, National Guard Health Affairs, Medina, SAU; 3 College of Medicine, Imam Abdulrahman Bin Faisal University, Dammam, SAU; 4 College of Medicine, University of Jeddah, Jeddah, SAU; 5 College of Pharmacy, Taibah University, Medina, SAU; 6 College of Medicine, Taibah University, Medina, SAU; 7 College of Medicine, Alrayan Colleges, Medina, SAU

**Keywords:** cancer, cancer-related pain, gastrointestinal cancer, gastrointestinal malignancy, opioid use

## Abstract

Background

Pain is a common symptom among individuals with gastrointestinal (GI) malignancies; yet, opioid use patterns and frequency remain understudied. Our study aims to characterize the frequency and patterns of opioid use among individuals with a history of GI malignancy diagnoses.

Methods

This study is a retrospective analysis of cross-sectional data from the 2019 and 2020 National Health Interview Survey (NHIS). A total of 594 adults with a previous diagnosis of GI cancer, namely, esophageal, stomach, pancreatic, liver, gallbladder, or colorectal cancers, were identified. Of these, 536 reported prescription medication use in the past 12 months and constituted the analytic population for opioid-use outcomes. Multivariate regression analysis with adjustment for sociodemographic and clinical covariates was used to identify factors associated with opioid use within the GI cancer cohort. As a secondary exploratory analysis using a different reference population (absence of each cancer type in the full cancer-history sample), we compared opioid use across all reported malignancy subtypes.

Results

Opioid use was reported by 143 out of the 536 respondents with a history of GI malignancy, who used prescription medications (26.67%), were younger (66.44 vs. 73.31 years, p<0.001), and were more likely to be smokers (87 (60.8%) vs. 193 (49.1%), p=0.02) compared with non-users. Among chronic users, 37 (46.6%) individuals reported daily use of opioids. Those with liver cancer and esophageal cancer had the highest rates of opioid use in the past 12 months (42.8% and 39.9%) and the lowest with colorectal malignancy (20.7%). Younger age and smoking history were independently associated with opioid use (adjusted odds ratio (aOR) 0.95 and aOR 1.61, respectively). Liver cancer (aOR 0.42) and colorectal cancer (aOR 0.52) were associated with lower odds of opioid use relative to other GI malignancy subtypes. In a separate exploratory model comparing opioid use across all reported cancer diagnoses, liver and esophageal cancers showed the strongest associations with opioid use (aOR 3.10 and aOR 2.85, respectively).

Conclusion

Opioid use is prevalent among GI cancer survivors and varies by malignancy subtype, with individuals diagnosed with esophageal and liver cancer showing the strongest association with opioid use, supporting consideration of cancer subtype-specific pain management strategies.

## Introduction

Gastrointestinal (GI) malignancies, including esophageal, gastric, hepatic, biliary, pancreatic, and colorectal cancers, account for a growing global burden. In 2022, GI cancers resulted in millions of cancer cases and deaths worldwide, and there is an expected increase in incidence over the coming decades, especially in lower-resource regions [1]. Further demonstrating this trend, population-based estimates show a significantly increased lifetime risk of both developing and dying from these malignancies at all levels of human development [2]. As diagnostic and therapeutic innovation increases life expectancy, the growing need for symptomatic management-particularly pain-has become fundamental in care for patients with GI malignancies.

Based on meta-analyzed data, more than half of the patients receiving active treatment and most of those with advanced disease report clinically significant pain, rendering it one of the most common and disturbing symptoms suffered by cancer patients [3].

Opioids play a central role in managing moderate to severe cancer-related pain. Opioid use in patients with GI cancers involves disease-specific considerations. Among patients with pancreatic cancer, opioid treatment is commonly initiated after diagnosis, although prescribing patterns differ according to demographic characteristics, geographic region, and the type of treatment received [4]. Likewise, opioid use is common following GI cancer surgery. Although many patients are opioid-naïve before surgery, a subset develops new persistent opioid use following surgery, which has been associated with poorer long-term health outcomes and increased mortality [5]. In addition to the dose and duration of opioid use, some studies have suggested that opioid exposure may be linked to poorer cancer outcomes, including lower survival rates, in patients with different types of GI cancer [6].

Along with the possible side effects, risk of dependence, and GI problems associated with opioids, these factors highlight the importance of understanding how opioids are prescribed and used among patients with GI malignancies. Previous studies have explored the patterns and prevalence of opioid use among various populations. However, data regarding its use in individuals with a history of GI malignancy remains limited. Our primary aim in this study is to characterize the patterns and frequency of prescription opioid use among adults with a self-reported history of GI malignancy using nationally representative data from the 2019-2020 NHIS. As a secondary, exploratory aim, we compared opioid use across all self-reported cancer diagnoses captured in the survey to contextualize GI-malignancy-specific findings within the broader landscape of cancer-related opioid use.

## Materials and methods

Study design and data source

This is a cross-sectional analysis of data from the 2019 and 2020 NHIS. The NHIS is a nationally representative survey that aims to gather data from non-institutionalized adults in the United States [7]. It is carried out annually and conducted throughout the year by the National Center for Health Statistics (NCHS). The Sample Adult component of the NHIS was used to draw the data in this study, in which a single adult is chosen from each sampled household to go through a complete interview and answer questions related to health, chronic conditions, and health-related behaviors. We combined the 2019 and 2020 surveys to increase the statistical power and stability of estimates in this study. Choosing these two years was based on the fact that they are the years in which the NHIS included the Prescription Opioid Use variable. Weighting, stratification, and clustering of variables were utilized in our study to produce nationally representative estimates. All procedures carried out in this survey were approved by the NCHS to ensure the production of high-quality data while protecting the participants’ privacy.

Study population

Our study combined data from the 2019 and 2020 NHIS; we collected sociodemographic variables, clinical variables, and cancer-related variables. In terms of cancer-related variables, participants with a self-reported history of GI malignancy were identified through a validated direct cancer item ("Have you EVER been told by a doctor or other health professional that you had cancer or a malignancy of any kind?") followed by a checklist of specific malignancy types. Those who were classified in our analysis as GI malignancy participants were those who confirmed having any of the following types of malignancy: esophageal, pancreatic, hepatic, gallbladder, gastric, rectal, colon, or colorectal cancer. Using this definition, we identified individuals with a history of GI malignancy, while the remaining respondents served as a comparison group. Participants with missing data on any covariate included in a given multivariable regression model were excluded from that specific analysis (complete-case analysis).

Outcome definition

The primary outcome of interest in this analysis was the patterns and frequency of opioid use, identified through several validated questions included in the 2019 and 2020 NHIS survey, administered in a skip-pattern sequence. First, all participants were asked, “At any time in the past 12 months, did you take prescription medication?” If they answered yes, they were asked a follow-up question: “During the past 12 months, have you taken any opioid pain relievers prescribed by a doctor, dentist, or other health professional? Examples include hydrocodone, Vicodin, Norco, Lortab, oxycodone, OxyContin, Percocet, and Percodan. If you are not sure, please tell me the name of the drug and I can look it up”. Those who reported opioid use in the past 12-month period were asked “During the past 3 months, did you take a prescription opioid to treat short-term or acute pain, such as pain due to a broken bone or muscle sprain, pain from dental work, or pain following surgery?”. Respondents who reported using opioids in the past three months were asked separately if they used it for an acute or chronic issue to assess how long they will keep using it. Frequency of use was evaluated through the question “During the past 3 months, how often did you take a prescription opioid? Would you say some days, most days, or every day?”, with answers categorized as “every day”, “most days”, or “some days”. Notably, only those who used opioids for a chronic condition were asked about the frequency of use.

Other covariates

Several sociodemographic and clinical covariates were gathered for descriptive and comparative analysis, including gender (male/female), age (continuous), educational level, body mass index (BMI, categorized as either underweight, healthy weight, overweight, or obese), and self-reported history of comorbid conditions such as hypertension, prediabetes, and diabetes. Tobacco use was another factor extracted in our study, and it included cigarette smoking, electronic cigarettes, cigars, and pipe smoking status.

Statistical analysis

To obtain nationally representative estimates and appropriate variance estimates, all analyses took into account the complex sample design of the NHIS, including the annual sampling weights, pseudo-strata, and pseudo-primary sampling units (PSUs). Survey design objects were constructed using the NHIS-supplied sampling weight, stratum, and primary sampling unit variables for the combined 2019-2020 Sample Adult files, in accordance with NCHS analytic guidelines for combining multiple survey years. GI malignancy subtypes and covariates were coded as binary indicators (presence/absence) based on the corresponding NHIS checklist items. Ninety-five percent (95%) confidence intervals (CIs) and survey-weighted percentages were presented with unweighted sample sizes (n). While categorical data were summarized as unweighted frequencies with weighted proportions and compared using the chi-square test, continuous variables were summarized using survey-weighted means with standard deviations and compared using the T-test.

For analyses of opioid-use patterns, denominators were defined according to the corresponding NHIS questionnaire skip patterns; all adults with a history of GI malignancy were included in the primary study population irrespective of prescription medication use.

Among participants with a history of GI malignancy, prescription medication use during the preceding 12 months was first assessed. Participants who reported prescription medication use during this period were subsequently classified according to whether they had used prescription opioids. Questions regarding opioid use during the past three months were evaluated among respondents eligible for those items based on the NHIS questionnaire skip pattern. Indications for opioid use were assessed among recent opioid users, whereas frequency of use was evaluated among participants who reported opioid use for chronic causes. Acute and chronic indications were not treated as mutually exclusive.

A survey-weighted multivariable logistic regression model was used to identify factors associated with prescription opioid use during the previous 12 months among adults with a history of GI malignancy. The model included age, sex, body mass index category, diabetes mellitus, hypertension, smoking status, and GI malignancy type. Because GI malignancy subtypes were not mutually exclusive, each subtype was entered as an independent binary indicator (presence vs. absence of that subtype) rather than as levels of a single categorical variable with one common reference group; adjusted odds ratios for each subtype therefore represent the independent association of that subtype with opioid use, conditional on the other subtypes and covariates in the model. Results are presented as adjusted odds ratios (aORs) with 95% CIs and corresponding P-values. Subpopulation analyses were performed while retaining the full complex survey design for variance estimation. Statistical significance was defined as a two-sided P < 0.05. Analyses were performed in Python version 3.12 within the Jupyter Notebook environment (Anaconda distribution). An exploratory model with Benjamini-Hochberg false discovery rate (FDR) correction was also applied in order to compare opioid use across all self-reported cancer diagnoses captured in the NHIS survey, using the absence of each respective cancer type within the full cancer-history population as the reference group. Because this reference population differs from that used in the primary GI-cohort model described above, adjusted odds ratios for the same cancer subtype are not directly comparable between the two models.

Ethical considerations

This study utilized NHIS surveys that are publicly available, de-identified secondary data, which were approved by the NCHS Research Ethics Review Board. Informed consent was obtained from all participants at the time of original data collection. This secondary analysis was exempt from additional institutional review due to its use of de-identified, publicly available data for research use.

## Results

Sample characteristics

Our study included 536 adults with a self-reported history of GI malignancies who are using prescription medications (Figure 1), namely, esophageal, stomach, pancreatic, liver, gallbladder, and colorectal cancers, collected from the 2019 and 2020 NHIS. A total of 143 individuals (26.67%, survey-weighted) reported opioid use, while the remaining 393 (73.33%) participants did not. In terms of age, opioid users were younger than those who don’t use opioids (66.44 ± 11.58 years vs. 73.31 ± 10.18 years, p<0.001) and were more likely to report a smoking history (87 (60.8%) vs. 193 (49.1%), p=0.02). There was no significant difference between the two groups in terms of gender (69 (48.3%) vs. 214 (54.5%) female, p=0.20), BMI category (p=0.37), diabetes mellitus (38 (26.6%) vs. 79 (20.1%), p=0.11), or hypertension (87 (60.8%) vs. 262 (66.7%), p=0.21). See Table 1.

**Figure 1:**
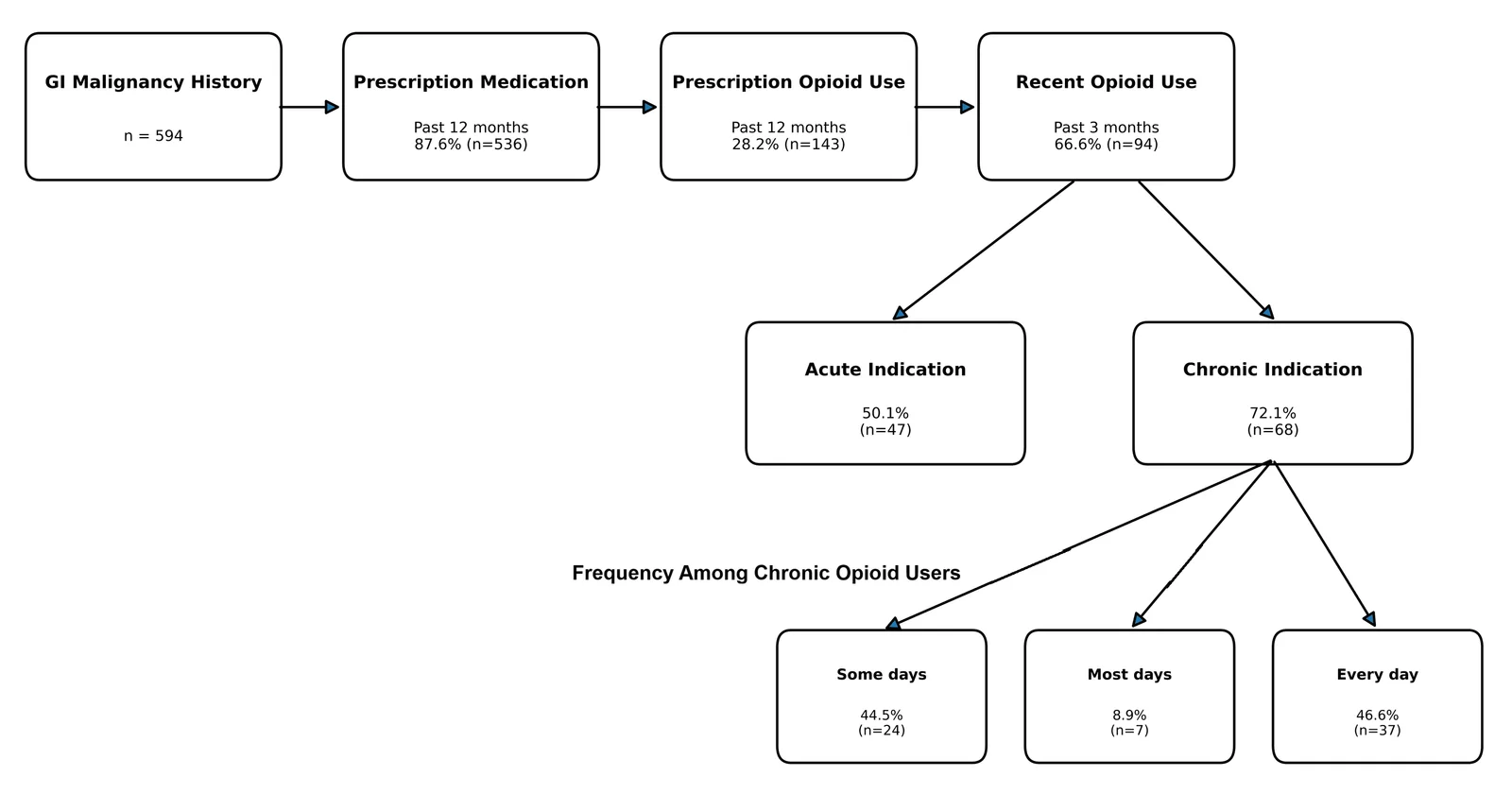
Prescription opioid use cascade among adults with a history of gastrointestinal malignancy (n = 594) Percentages represent survey-weighted prevalence estimates and were calculated using the National Health Interview Survey (NHIS) annual sampling weights while accounting for the complex survey design, including pseudo-strata and clustering by pseudo-primary sampling units (PSUs). Accordingly, denominators varied across stages because of the NHIS questionnaire skip patterns. Past-3-month opioid use was assessed among past-12-month opioid users; acute and chronic indications were evaluated among recent opioid users; and frequency of use was assessed among participants reporting opioid use for chronic causes. Acute and chronic indications were not mutually exclusive. Unweighted participant counts are shown alongside the corresponding survey-weighted prevalence estimates.

**Table 1:** Characteristics of adults with a history of gastrointestinal malignancy who reported prescription medication use in the past 12 months Categorical variables are presented as unweighted frequencies (n) with survey-weighted percentages, and continuous variables are presented as survey-weighted means with corresponding standard deviations, as appropriate. Estimates incorporated the National Health Interview Survey (NHIS) sampling weights, strata, and primary sampling units to account for the complex survey design. Gastrointestinal malignancies included colorectal, esophageal, stomach, pancreatic, liver, and gallbladder cancers. Percentages may not total 100% because of rounding or missing responses. Categorical variables were compared using the Chi^2^ test, and continuous variables were compared using the t-test. Of 594 participants, only 536 received a prescription for medication in the last 12 months.

Variable	Total		No opioid use	Opioid use	Statistic values	p-value
Number (%)	536		393 (73.33)	143 (26.67)		
Gender (Female, n (%))	283 (52.8)		214 (54.5)	69 (48.3)	Chi2 = 1.62	0.2
Age (years, mean ± SD)	70.89 ± 11.37		73.31 ± 10.18	66.441 ± 11.58	T value = -6.69	<0.001
BMI Category						
Underweight (Yes, n (%))	12 (2.2)		8 (2.0)	4 (2.8)	Chi2 = 3.16	0.37
Healthy weight (Yes, n (%))	165 (30.8)		126 (32.1)	39 (27.3)		
Overweight (Yes, n (%))	182 (34.0)		137 (34.9)	45 (31.5)		
Obese (Yes, n (%))	177 (33.0)		122 (31.0)	55 (38.5)		
History of smoking (Yes, n (%))	280 (52.2)		193 (49.1)	87 (60.8)	Chi2 = 5.78	0.02
Diabetes mellitus (Yes, n (%))	117 (21.8)		79 (20.1)	38 (26.6)	Chi2 = 1.57	0.11
Hypertension (Yes, n (%))	349 (65.1)		262 (66.7)	87 (60.8)	Chi2 = 2.57	0.21

Opioid use patterns and frequency

Among individuals reporting chronic opioid use, the frequency distribution was as follows: some days, 24 (44.5%, 95% CI 30.8-59.1); most days, 7 (8.9%, 95% CI 3.8-19.7); and every day, 37 (46.6%, 95% CI 32.8-60.9). Notably, nearly half of chronic opioid users reported an everyday use pattern (Table 2). Opioid use in the past 12 months was the highest among individuals who reported a previous diagnosis of liver cancer (42.8%), followed by esophageal cancer (39.9%), pancreatic cancer (32.5%), gallbladder cancer (32.5%), and stomach cancer (31.3%), and was the lowest among individuals with a previous diagnosis of colorectal cancer (20.7%). As for opioid use in the past 3 months, a similar pattern was observed, where the highest rate of opioid use was noted among individuals with a diagnosis of esophageal cancer (38.4%) and liver cancer (35.8%), and the lowest for individuals with gallbladder cancer (6.3%). Likewise, chronic opioid use was mostly observed in participants with a self-reported history of esophageal and liver cancer (32.3% and 26.2%, respectively). Similarly, everyday use was noted to have the highest rate among esophageal cancer individuals (19.4%), followed by individuals with pancreatic cancer (14.4%) (Figure 2).

**Table 2:** Frequency of prescription opioid use among adults reporting opioid use for chronic pain (n = 68) Values are presented as unweighted frequencies (n) and survey-weighted percentages with 95% confidence intervals. The denominator for all percentages was the total number of adults with a history of gastrointestinal malignancy who reported prescription opioid use for chronic pain and had a valid response to the frequency-of-use question (n = 68). Survey-weighted percentages were calculated by dividing the sum of the National Health Interview Survey (NHIS) sampling weights for participants in each frequency category by the sum of the sampling weights for all 68 eligible participants and multiplying by 100. Corresponding 95% confidence intervals accounted for the NHIS sampling weights, strata, and primary sampling units.

Frequency	N (%)	95% CI
Some days	24 (44.5)	(30.8–59.1)
Most days	7 (8.9)	(3.8–19.7)
Every day	37 (46.6)	(32.8–60.9)

**Figure 2:**
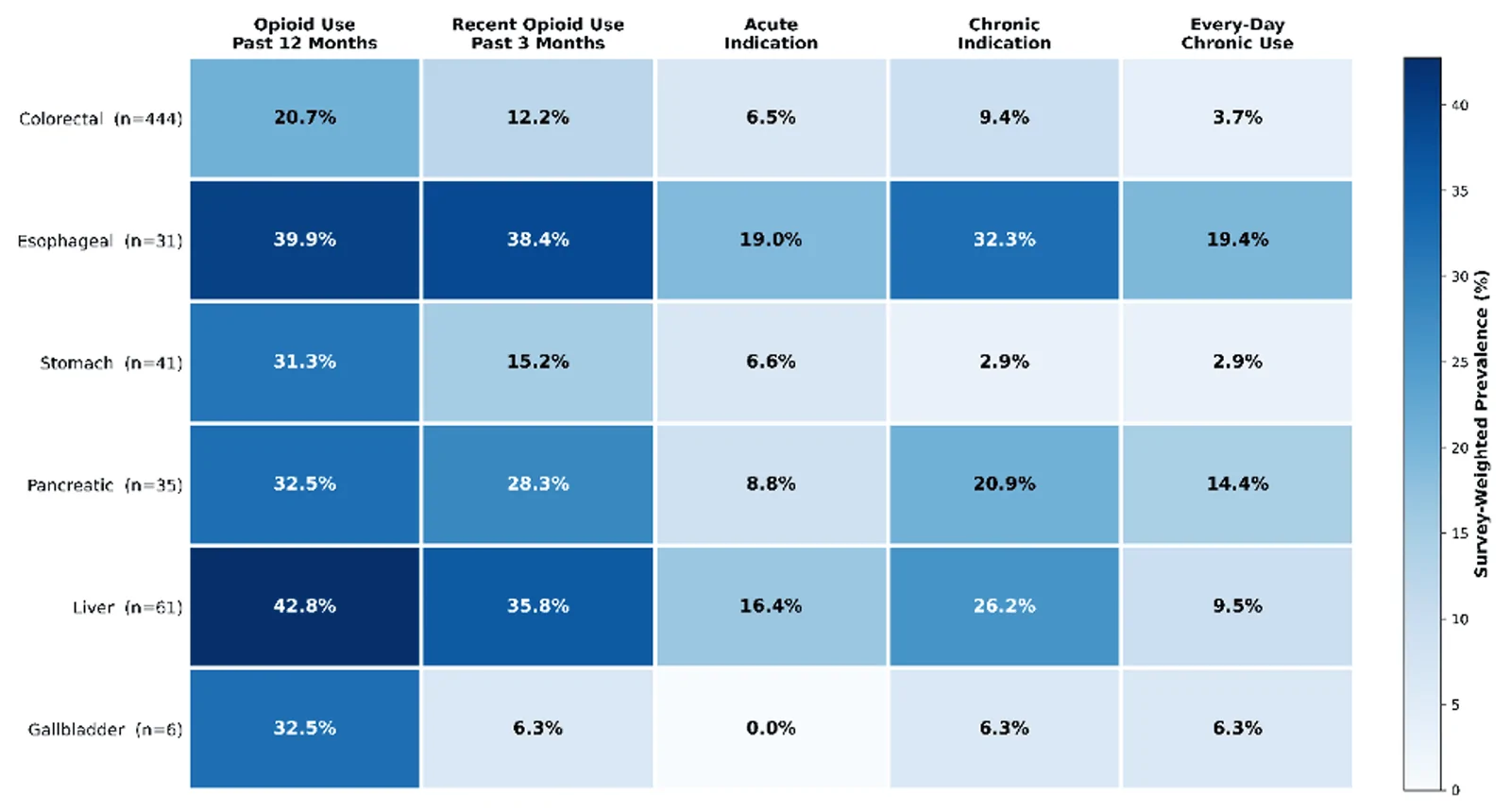
Cancer-specific patterns of prescription opioid use among adults with a history of gastrointestinal malignancy Values in each box represent survey-weighted percentages. For each gastrointestinal cancer type, the denominator was the total number of participants with a history of that specific malignancy, allowing direct comparison of opioid-use patterns across cancer types. The percentages therefore represent the weighted proportion of participants with each cancer type who reported prescription opioid use in the past 12 months, use in the past 3 months, an acute indication, a chronic indication, or daily use among chronic opioid users, as applicable. Acute and chronic indications were not mutually exclusive; participants could report opioid use for both acute and chronic reasons. Consequently, the 47 participants reporting an acute indication and the 68 reporting a chronic indication should not be summed as independent groups, as only 94 participants reported opioid use in the past 3 months. All percentages accounted for the National Health Interview Survey (NHIS) sampling weights, strata, and primary sampling units.

Opioid use phenotypes

In our study sample, 94 individuals reported using opioids in the past 3 months. Among these, chronic-only use of opioids was the most prevalent phenotype (44.4%), followed by acute-plus-chronic use (27.7%), acute-only (22.3%), and neither of which (5.5%). Individuals with pancreatic and esophageal malignancies showed predominance in chronic-only use (61.8% and 50.6%), followed by the liver cancer (48.8%) and colorectal cancer (46.9%) subgroups. See Figure 3.

**Figure 3:**
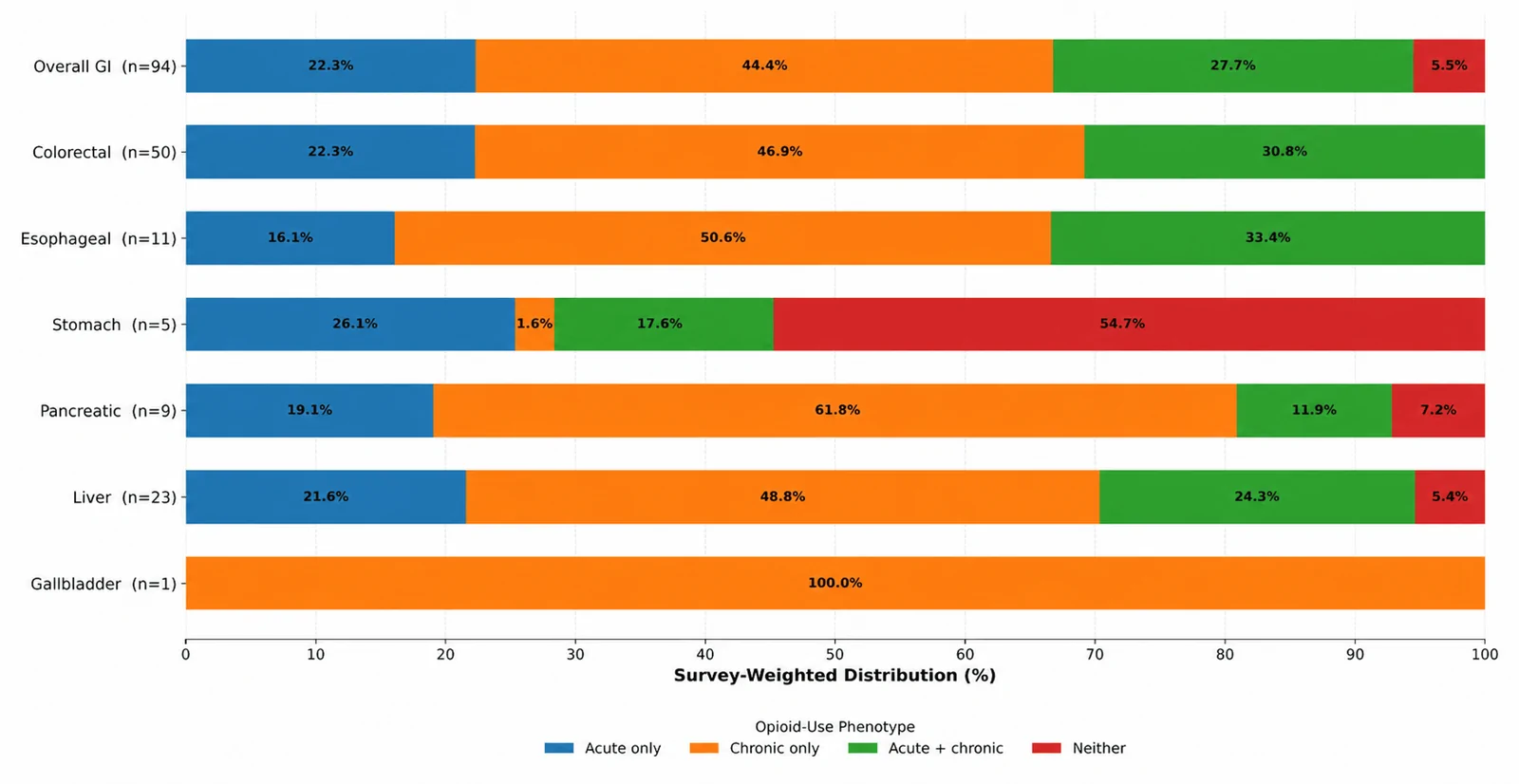
Acute and chronic prescription opioid-use phenotypes among adults with a history of gastrointestinal malignancy reporting prescription opioid use in the past 3 months (n = 94) Bars represent the survey-weighted distribution of mutually exclusive opioid-use phenotypes: acute only, chronic only, both acute and chronic, and neither acute nor chronic. For the overall gastrointestinal malignancy group, the denominator was the 94 participants who reported prescription opioid use during the past 3 months and had valid responses to the acute- and chronic-indication questions. For each cancer-specific row, the denominator was the number of eligible past-3-month opioid users with a history of that specific malignancy, as shown in parentheses beside the cancer type. Survey-weighted percentages were calculated by dividing the sum of the National Health Interview Survey (NHIS) sampling weights for participants in each opioid-use phenotype by the sum of the sampling weights for all eligible participants in the corresponding row and multiplying by 100. Thus, the percentages within each row sum to approximately 100%. Acute and chronic indications were originally non-mutually exclusive; for this figure, they were combined into mutually exclusive categories of acute only, chronic only, acute plus chronic, and neither. Because participants could have a history of more than one gastrointestinal malignancy, cancer-specific row counts are not mutually exclusive and should not be summed to obtain the overall sample size. Survey estimates accounted for the NHIS sampling weights, strata, and primary sampling units.

Factors associated with opioid use

Multivariate logistic regression analysis was utilized among the GI cancer cohort with adjustment for age, gender, BMI, diabetes, hypertension, smoking, and GI cancer subtypes. Building on the descriptive findings presented above, the following results reflect adjusted associations from multivariable regression models rather than unadjusted comparisons. Increasing age was found to be independently associated with lower odds of opioid use (aOR 0.95 per year, 95% CI 0.93-0.97, p<0.001), and smoking history was associated with higher odds of opioid use (aOR 1.61, 95% CI 1.06-2.87, p<0.001). A self-reported history of colorectal malignancy and liver malignancy were each associated with lower odds of opioid use relative to other GI cancer subtypes (aOR 0.52, 95% CI 0.33-0.81, p=0.004 and aOR 0.42, 95% CI 0.24-0.80, p=0.004, respectively). Other factors, including gender, BMI, diabetes, hypertension, and history of other GI malignancy subtypes such as stomach, pancreatic, esophageal, or gallbladder cancer, were not significantly associated with opioid use among individuals with a previous diagnosis of GI malignancy. See Table 3.

**Table 3:** Multivariate logistic regression analysis of factors associated with opioid use among patients with GI malignancies (n = 536) Values are presented as adjusted odds ratios (aORs) with 95% confidence intervals (CIs). The survey-weighted multivariable logistic regression model accounted for National Health Interview Survey (NHIS) sampling weights, strata, and primary sampling units. The outcome was prescription opioid use during the past 12 months. The model was adjusted for age, sex, body mass index, diabetes mellitus, hypertension, smoking status, and gastrointestinal malignancy type. Cancer types were entered as independent binary indicators rather than as levels of a single categorical variable, because subtypes were not mutually exclusive (see Statistical Analysis). The overall sample size included in this analysis of participants who received a prescription in the past 12 months is 536 out of 594, with Snell and Cox R² = 0.115.

	aOR	CI	p-value
Age	0.95	0.93 - 0.97	<0.001
Gender			
Female	Reference		
Male	0.85	0.56 - 1.30	0.44
BMI			
Healthy weight	Reference		
Underweight	1.78	0.42 - 2.1	0.43
Overweight	0.99	0.58 - 1.79	0.96
Obese	1.61	0.67 - 2.03	0.59
History of smoking			
None	Reference		
Yes	1.61	1.06 - 2.87	<0.001
Diabetes mellitus			
None	Reference		
Yes	1.51	0.91 - 2.50	0.11
Hypertension			
None	Reference		
Yes	0.89	0.57 - 1.38	0.60
Colorectal cancer			
None	Reference		
Yes	0.52	0.33 - 0.81	0.004
Stomach cancer			
None	Reference		
Yes	0.85	0.38 - 1.91	0.69
Pancreatic cancer			
None	Reference		
Yes	0.69	0.31 - 1.57	0.38
Esophageal cancer			
None	Reference		
Yes	0.62	0.27 - 1.41	0.25
Liver cancer			
None	Reference		
Yes	0.42	0.24 - 0.80	0.004
Gall bladder cancer			
None	Reference		
Yes	0.30	0.05 - 1.91	0.20

Cross-cancer comparison

A broader model comparing opioid use across all cancers reported in the survey population (adjusted for age, gender, BMI, diabetes, hypertension, and other cancer diagnoses, and with the application of Benjamini-Hochberg false discovery rate (FDR) correction) was utilized for the purpose of this study (Figure 4). In this model, a self-reported history of diagnosis with liver cancer was independently associated with the highest adjusted odds of opioid use among all 23 reported cancer subtypes (aOR 3.10, 95% CI 1.50-6.38, p=0.002, FDR q=0.027), remaining significant after multiplicity correction. A previous diagnosis of esophageal cancer showed a similarly elevated point estimate (aOR 2.85, 95% CI 1.21-6.71, p=0.017), but its FDR-corrected q-value (0.099) did not meet the significance threshold of 0.05. Other cancers, including pancreatic and stomach cancers, demonstrated similar magnitude but did not achieve statistical significance (aOR 2.08, 95% CI 0.90-4.78, p=0.085 and aOR 2.05, 95% CI 0.80-5.28, p=0.137, respectively). Furthermore, colorectal cancer was not shown to be significantly associated with opioid use in the corrected model (aOR 1.19, 95% CI 0.85-1.66, p=0.317). Of note, liver and colorectal cancers showed different directions of association between the model that examined opioid use among the GI cancer cohort and the one examining opioid use across all cancers, which implies the different reference populations used in each analysis (GI cancer subtypes vs. absence of the specific cancer type in the full cancer history population).

**Figure 4:**
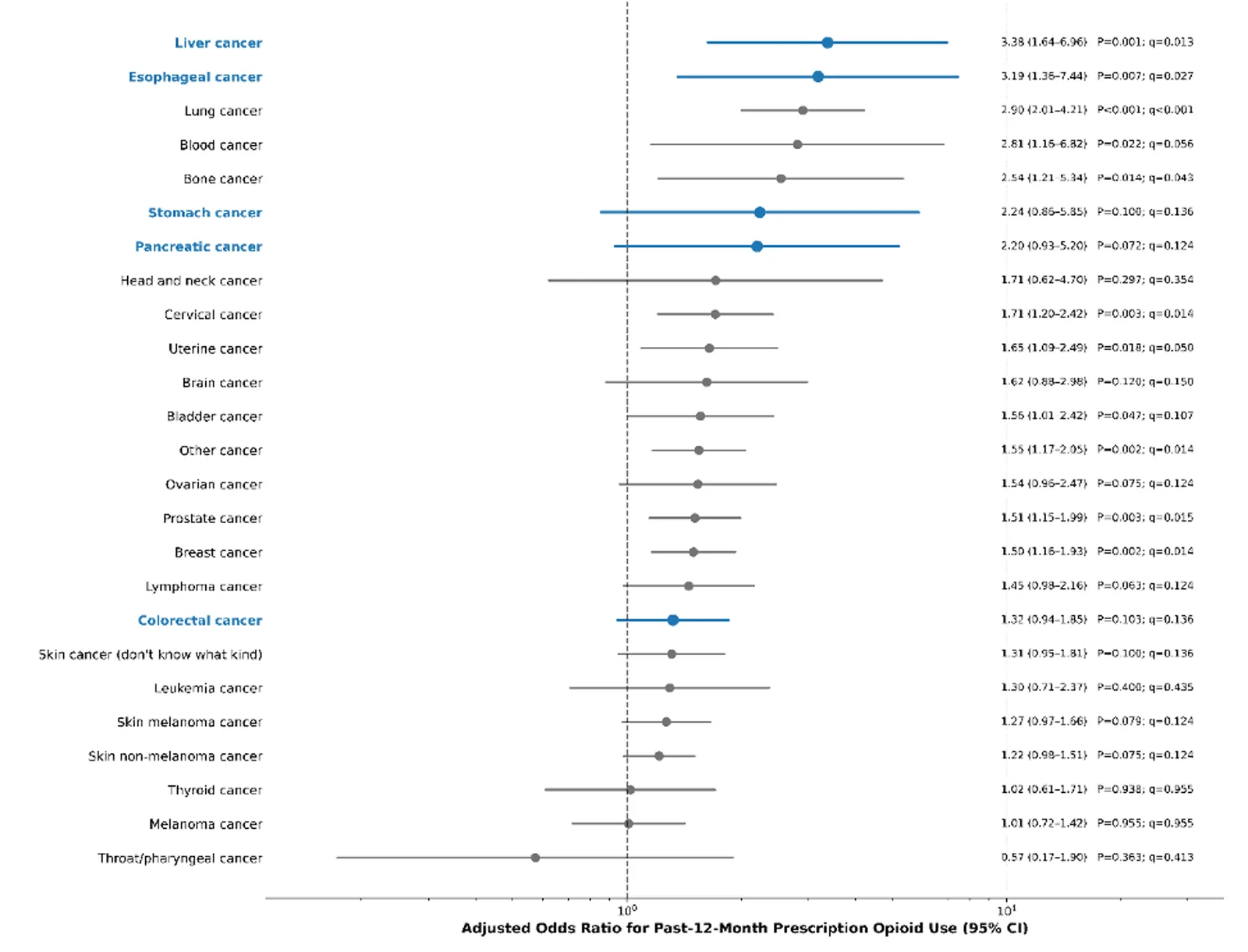
Multivariate logistic regression of factors associated with prescription opioid use among adults with a history of gastrointestinal malignancy (n = 594) Adjusted odds ratios (aORs) and 95% confidence intervals (CIs) were estimated using a survey-weighted multivariable binary logistic regression model accounting for the National Health Interview Survey (NHIS) sampling weights, strata, and primary sampling units. The outcome was prescription opioid use during the past 12 months. The model included age, sex, body mass index category, diabetes mellitus, hypertension, smoking status, and gastrointestinal malignancy type. The analytical sample included 594 participants with complete data for all variables included in the model. The vertical reference line represents an odds ratio of 1.0.

## Discussion

In this retrospective analysis of2019-2020 NHIS data, about one in four adult individuals who received a previous diagnosis of GI malignancy reported opioid use. Hepatic and esophageal malignancy histories had the highest prevalence and adjusted odds of opioid use, with approximately half of those determined to be chronic users reporting using opioids every day. Factors associated with opioid use among GI malignancy individuals included younger age and smoking history, while those with colorectal cancer exhibited lower odds of opioid use compared with other malignancies after adjustment. The observations in our analysis extend a small but growing body of literature that examined patterns of opioid use among individuals with GI malignancies, thus highlighting cancer subtypes as an important, but underappreciated, factor affecting the likelihood of opioid use.

The finding of opioid use being highly prevalent among individuals with liver cancer aligns with the descriptions in the literature of hepatocellular carcinoma (HCC) pain as one of the most prevalent and undertreated symptoms in oncology, mainly caused by invasive processes of the primary or metastatic lesion, hepatic capsular irritation, or because of liver cirrhosis [8]. This comes as a paradox, as HCC simultaneously poses challenges when it comes to analgesic use to manage pain due to a narrowed therapeutic window due to impaired hepatic clearance and the risk of drug accumulation and encephalopathy, with concurrent misconceptions held by the patients about analgesic safety in liver disease, resulting in undertreatment independent of the actual liver function [9]. This conflict between misconceptions of the augmented risk of drug toxicity and increased symptom burden may explain why liver cancer demonstrated the highest prevalence of opioid use in the unadjusted model and the strongest adjusted association with opioid use after adjustment. Noteworthy, the observation of liver cancer being associated with lower odds of opioid use relative to other GI subtypes (in the GI-only model) might be a result of differing reference populations rather than contradictory findings. However, we cannot rule out that unmeasured differences in treatment intensity or surgical approach across subtypes contribute to this association.

Our findings of esophageal cancer individuals having an increased opioid burden likely reflect the bothersome symptom profile associated with this subtype of malignancy, including dysphagia-associated nutritional deficits and the morbidity associated with esophagectomy and multimodal therapy, which may contribute to persistent post-treatment chronic pain. As regards opioid use among pancreatic cancer individuals, the findings in our cohort (32.5%) are lower than clinical-cohort estimates, which suggest that up to 80% of pancreatic cancer patients experience pain and up to half of them require strong opioids as analgesics [4,10]. This discrepancy likely results from differences in populations, as our study uses data from individuals at different stages and times since diagnosis, compared with cohorts drawn from settings with active, often advanced, disease. Comparably, the relatively lower burden of opioid use among colorectal cancer individuals reflects the results reported in the survivorship literature, which describes a shift towards bowel dysfunction symptoms (e.g., diarrhea, constipation) rather than pain as the major late symptoms in this population [11].

The inverse association observed in our study between age and opioid use is consistent with patterns of declining opioid utilization among older individuals with cancer reported in the literature, a trend that is likely attributable to caution surrounding renal and hepatic clearance, risk of falls, and polypharmacy in the elderly, as well as the effects of opioid stewardship policies including the 2016 CDC opioid prescribing guidelines [12,13]. Notably, this pattern has been linked in the literature with increased pain-related emergency department visits among elderly patients with cancer [14]. Therefore, this raises the possibility that our findings reflect undertreatment rather than lower needs for strong analgesics.

The association between smoking and opioid use correlates with evidence in the literature linking tobacco use to higher chronic pain burden, opioid dosing, and risk of opioid use disorder. This is possibly mediated by nicotine-opioid cross-tolerance and nicotine-withdrawal hyperalgesia [15,16]. Furthermore, higher odds of developing opioid use disorder were found with current tobacco users in chronic pain populations [16]. Thus, smoking status represents a clinically important marker for risk stratification when opioids are considered as a main-line management option for cancer-associated pain in GI malignancy survivors.

With regard to the frequency of opioid use, our analysis found that approximately half of chronic opioid users reported using opioids daily, which highlights the importance of close monitoring of persistent opioid use in this population. However, longitudinal analyses of older cancer survivors with non-malignant pain have established that only one in eight individuals continue opioid use over extended periods [17]. Our cross-sectional study, nevertheless, cannot establish long-term trajectories to confirm whether or not our population cohort follows the same path in the long term, which represents an important direction for future, well-designed longitudinal studies.

Our study has several strengths. First, the use of NHIS data with appropriate use of sample weights and stratification variables allows generalization of the observations to the population of adults with a history of GI malignancy in the United States, in contrast to single-institution or clinical cohort studies, which might introduce biases. Second, the characterization of GI cancer subtypes rather than including GI malignancies as a single, homogeneous group allows the analysis to capture clinically relevant data regarding the opioid use burden that would be obscured in pooled analyses. Third, adjusting for multiple variables and including a cross-cancer comparison model with false discovery rate correction strengthens confidence in the aforementioned findings and helps contextualize cancer subtype-specific findings against the broader picture of cancer-related opioid use.

However, several limitations in our analysis warrant consideration. First, the cross-sectional nature of NHIS-based studies cannot establish temporality or causality. Thus, this study precludes temporal or causal association between cancer diagnosis and opioid initiation. Second, opioid use, cancer diagnosis, and comorbidities were self-reported, which subjects these variables to recall and misclassification bias. Third, some cancer subtypes have limited population size, such as gallbladder cancer, which limits the precision of estimates and limits the ability to analyze data related to the respective cancer subtype. NHIS data does not specify cancer stage, treatment received, surgical approach (e.g., open vs. minimally invasive resection, or non-operative management), recurrence status, or time since diagnosis--all of which strongly influence pain trends and opioid use. In particular, procedures such as open hepatectomy or esophagectomy possibly carry a higher risk of chronic post-surgical pain than minimally invasive or conservative management. Because these factors cannot be taken into account, the subtype-specific associations we observed (e.g., for liver and esophageal cancer) may partly reflect differences in treatment intensity across subtypes rather than the intrinsic property of the malignancy itself. Importantly, the survey cannot confirm whether reported opioid use was indicated specifically for malignancy-related pain, as opposed to an unrelated acute or chronic condition, which limits the clinical specificity of our findings. Also, the limited number of covariates that can be explored and included in the analysis could affect the results in this study. Therefore, future, well-structured longitudinal studies are needed to further explore the association between GI-cancer subtypes and opioid use.

## Conclusions

In conclusion, this nationally representative analysis demonstrated that opioid use is prevalent among individuals with a history of gastrointestinal malignancy, affecting approximately one in four individuals, with about half of chronic users reporting daily use. Burden differed by cancer subtype, with liver and esophageal cancer individuals showing the highest use, while colorectal cancer individuals demonstrated the lowest. Age and smoking history were independently associated with opioid use. These subtype-specific differences may reflect variation in disease and treatment burden that warrants further investigation, and, pending confirmation in studies with detailed clinical and treatment data, could inform consideration of subtype-specific pain management strategies.
